# Molecular study of the presence and transcriptional activity of HPV in semen

**DOI:** 10.1007/s40618-023-02167-4

**Published:** 2023-08-16

**Authors:** F. Faja, F. Pallotti, S. Bianchini, A. Buonacquisto, G. Cicolani, A. C. Conflitti, M. Fracella, E. N. Cavallari, F. Sciarra, A. Pierangeli, D. Paoli, A. Lenzi, G. Antonelli, F. Lombardo, D. Gianfrilli

**Affiliations:** 1 Laboratory of Seminology - “Loredana Gandini” Sperm Bank, Department of Experimental Medicine, “Sapienza” University of Rome, 00161 Rome, Italy; 2 Laboratory of Microbiology and Virology, Department of Molecular Medicine, “Sapienza” University of Rome, 00185 Rome, Italy; 3 Section of Medical Pathophysiology and Endocrinology, Department of Experimental Medicine, “Sapienza” University of Rome, 00161 Rome, Italy; 4 Faculty of Medicine and Surgery, University of Enna “Kore”, 94100 Enna, Italy

**Keywords:** Human Papillomavirus, Spermatozoa, Semen quality, Risk factors, Detection limit, Transcriptional activity

## Abstract

**Purpose:**

Human Papillomavirus (HPV) in semen represents a controversial topic. Recent evidence suggests a correlation with poor semen quality, but its detection is still unstandardized in this biological fluid. Thus, the aims of this study were to verify the ability of nested PCR to reveal HPV-DNA in semen; to evaluate association of seminal HPV with sperm parameters and risk factors for infection; to investigate the rate of HPV-DNA positivity in patients with and without risk factors; to assess HPV transcriptional activity.

**Methods:**

We enrolled sexually active men and collected clinical and anamnestic data during andrological and sexually transmitted infections (STIs) evaluation. For each patient, we performed semen analysis and nested PCR to detect HPV-DNA in semen. In positive semen samples, we proceeded with genotyping and RNA quantification to detect HPV transcriptional activity.

**Results:**

We enrolled 185 men (36.0 ± 8.3 years), of which 85 with (Group A) and 100 without HPV risk factors (Group B). Nested PCR was able to reveal HPV-DNA in semen, discovering a prevalence of 8.6% (11.8% in Group A and 6% in Group B, respectively). We observed no correlation between sperm quality and seminal HPV. Genital warts and previous anogenital infection were significantly associated with the risk of HPV positivity in semen. Moreover, no viral transcriptional activity was detected in positive semen samples.

**Conclusions:**

Our study suggests that searching for seminal HPV could be important in patients both with and without risk factors, especially in assisted reproduction where the risk of injecting sperm carrying HPV-DNA is possible.

**Supplementary Information:**

The online version contains supplementary material available at 10.1007/s40618-023-02167-4.

## Introduction

Sexually Transmitted Infections (STIs) represent a global health issue as they cause acute and chronic diseases that can lead to gestational complications and infertility [1].

STIs are caused by bacteria, protozoa and viruses which colonize the genital tract leading to an inflammatory state and a consequent increase of oxidative stress potentially harmful to spermatogenesis [2]. This hypothesis is confirmed by the observation that pathogens in semen have been associated with alterations of sperm parameters [3–6].

One of the major sexually transmitted viruses is Human Papillomavirus (HPV), a non-enveloped double stranded DNA virus with tropism for cutaneous and mucosal epithelia. Infections with low-risk HPV genotypes (LR-HPVs) result in benign diseases, such as genital warts or papillomas, which can persist for months or years, but which are generally resolved by the host’s immune system. Conversely, persistent infection with high-risk types (HR-HPVs) can promote the development of tumors in the cervix, vulva and vagina in women, cancer of the penis in men and tumors in the anal canal and the oropharynx in both genders.

Seminal HPV infection can represent an important reproductive concern, as demonstrated by evidence showing a correlation with a poor semen quality, especially in regards of sperm motility [7–13]. Furthermore, anti-sperm antibodies (ASAs), induced by the presence of HPV on the sperm surface [14], could also contribute to male infertility [15, 16].

HPV infection seems to have an alleged harmful effect on embryonic development, probably resulting in a premature trophoblast degeneration, which could explain the increased rate of miscarriages reported in both natural and assisted pregnancies [17–20].

However, although the presence of HPV in semen may be clinically important in relation to male fertility, such as to assisted reproductive techniques (ART), to date its detection in this biological fluid is not widespread and it is still unstandardized. Moreover, it is not yet known whether its presence could be associated with viral activity.

Generally, in biological samples HPV viral load can be measured with different techniques relying on specific molecular mechanisms, each one carrying a variety of benefits and limitations. The main methods used for HPV detection and genotyping are hybridization assays and nucleic acid amplification. In situ hybridization techniques are direct methods which allow HPV localization within specific lesions but require high amounts of viral DNA; hence, samples with lower amounts of HPV-DNA can result falsely negative. To overcome this sensitivity limit, signal amplification DNA-based assays were developed and enable the detection of lower viral DNA by amplifying the signal emitted by the probe. However, this higher sensitivity is counterbalanced by a possible cross-reactivity between the probes and HPV types not included in the probe mix, which lowers the specificity of the method.

In alternative, nucleic acid amplification techniques can be used. Real Time PCR offers the advantage of performing amplification and detection simultaneously but requires a control for amplification efficiency and sample inhibition. In fact, quantity of HPV-DNA could be underestimated due to various factors, such as high sample DNA that may interfere with primer and probe binding to target DNA, poor quality of sample DNA, presence of inhibitors of PCR in anogenital secretions which reduce efficiency of amplification and/or HPV polymorphisms that reduce binding of primers or probes to target sequences impairing amplification. PCR and nested PCR show high analytical sensitivity detecting low viral load at the expense of specificity. Moreover, contamination and false-positive results are possible if good lab practices are not respected. Nevertheless, nucleic acid amplification techniques are considered ideal methods in HPV testing since they minimize misclassification of viral infection and allow detection of lower HPV-DNA.

Therefore, in the light of the scant literature evidence and the aforementioned diagnostic limitations, the aims of our study were to:

- verify the ability and the detection limit of the nested polymerase chain reaction (nested PCR) to reveal HPV-DNA in human semen;
- evaluate the association between the possible presence of seminal HPV and risk factors for infection;
- investigate the rate of HPV-DNA positivity in semen of patients with and without risk factors for HPV infection;
- assess HPV transcriptional activity in semen;
- describe semen parameters and ASAs presence in HPV-positive semen samples.

## Materials and methods

### Patients

The study was approved by our University Hospital’s Institutional Review Board (Ethical Committee of “Sapienza” University of Rome—Azienda Ospedaliera Policlinico Umberto I, Ref 6564, Prot. 0044/2022) and all patients gave their informed written consent.

We enrolled consecutive Caucasian men between January 2022 and June 2022, which were sexually active and over 18 years of age, attending the Laboratory of Seminology—Sperm Bank “Loredana Gandini”, Department of Experimental Medicine at “Sapienza” University of Rome, for andrological evaluation, and the Infectious Disease Department of the Policlinico Umberto I Hospital—“Sapienza” University of Rome, for STIs assessment.

For each subject, the following clinical and anamnestic data were collected by a detailed questionnaire during the andrological and STIs evaluation: previous andrological and non-andrological pathologies, anthropometric data (height, weight, Body Mass Index “BMI”), lifestyle (such as current smoking and alcohol drinking), sexual behaviors (stable sexual inter-courses with the same partner or promiscuous sexual intercourses with a number of partners > 1), previous and/or current urogenital tract infections, anti-HPV vaccination, any previous and/or current HPV infection of the patient and sexual partner. In particular, we referred to a personal history of anogenital HPV infection as an anogenital infection of the patients enrolled caused by HPV, diagnosed following epithelial scraping of external genitalia, either currently present or previously treated. We also investigated the history of anogenital HPV infection of their sexual partners considering patients with a partner with an HPV infection diagnosed within 12 months.

Men taking any medications (antibiotics, anabolic hormones) and/or with medical conditions associated with impaired semen parameters (endocrine diseases, testicular trauma, clinically relevant varicocele, cryptorchidism, testicular or other cancer, previous chemotherapy and/or radiotherapy, Klinefelter syndrome and other chromosome abnormalities or genetic syndromes) were excluded from the study.

Based on clinical data and on medical history, the enrolled subjects were divided into two study groups: Group A, which includes patients with risk factors for HPV infection (unprotected sexual intercourses with multiple partners, partners with HPV infection diagnosed within 12 months, personal history of anogenital HPV infection and/or genital warts), and Group B, which comprises subjects with no known risk factors for HPV infection (Table S1).

### Semen analysis and study of anti-sperm immunity

Semen samples were collected by masturbation after 3–5 days of abstinence. All samples were allowed to liquefy at 37 °C for 60 min and were then assessed according to World Health Organization “WHO” 2010 [21]. The following variables were taken into consideration: ejaculate volume (ml), sperm concentration (10^6^ per ml), total sperm number (10^6^ per ejaculate), progressive motility (%) and morphology (% abnormal forms).

To assess the possible presence of ASAs, in each semen sample we performed direct SpermMar Test (FertiPro N.V., Beernem, Belgium), evaluating the percentage of motile sperm that presented latex particles (coated with human IgG or IgA) bound and the site of the bond (head, midpiece, tail). Positivity was defined as the SpermMar Test showing binding > 20%, but clinical relevance was considered with a binding  percentage  > 50% [22].

### Verification of feasibility and detection limit of the molecular methodology

To evaluate the feasibility of HPV-DNA detection in seminal fluid with nested PCR, a semen sample with WHO parameters within 25th percentile collected by a patient with a negative history of HPV-DNA detection and physical examination for HPV infection (negative control) was spiked with an inactivated HPV-16 pellet (Helix Elite™ Molecular Standards).

In particular, the lyophilized pellet, containing 100.000 copies of inactivated HPV-16, was rehydrated with 200 µl of sterile nuclease-free water, following the manufacturer’s instructions.

The negative control sample was divided into 10 aliquots of 200 µl and the first one was spiked with the rehydrated pellet (200 µl), proceeding with serial dilutions 1:2 to achieve seminal aliquots containing 50.000, 25.000, 12.500, 6.250, 3.125, 1.562, 781, 391, 195 and 98 HPV copies, respectively.

All aliquots were processed for total DNA extraction and HPV-DNA detection, performing the same molecular techniques used for semen samples of the recruited subjects.

### Total DNA extraction

DNA extraction from aliquots of total semen samples (200 µl) was performed by QIAamp DNA mini kit (Qiagen, Milan, Italy), according to the manufacturer’s instructions. Extracted DNA was quantified by NanoDrop ND-2000 (Thermo Fisher Scientific, Waltham, MA, USA) and underwent molecular analysis to detect HPV-DNA.

### HPV-DNA detection in semen samples

For each sample, the presence of amplifiable DNA was tested by qualitative PCR using HLA1/HLA2 primers, which are specific for a highly conserved region of a human gene belonging to the family coding for the HLA complex. The presence of HPV was then investigated by nested PCR using MY09/MY11 as outer primers [23] and GP5 + /GP6 + as inner primers [24], which are specific for the viral gene coding capsid protein L1 (Table S2).

The amplification reaction with HLA1/HLA2 and MY09/MY11 primers was carried out using 100 ng of DNA in 25 μl under the following PCR conditions: 95 °C for 2 min followed by 35 cycles at 95 °C for 45 s, 54 °C for 1 min, 72 °C for 1 min and a final extension step at 72 °C for 7 min. The nested PCR with GP5 + /GP6 + primers was carried out in 25 μl using 1 μl of the first-round product under the following PCR conditions: 94 °C for 2 min followed by 30 cycles at 94 °C for 30 s, 45 °C for 30 s, 72 °C for 20 s and a final extension step at 72 °C for 5 min [25]. In each amplification reaction, DNA from a semen sample of a patient with negative history and physical examination for HPV infection was used as negative control, while the same DNA spiked with an inactivated HPV-16 pellet was used as positive control.

A 5 μl of each PCR product was then used for electrophoresis on 1.5% agarose gel to check the presence and exact length of the amplified fragments (230 bp, 450 bp and 150 bp for PCR products obtained with HLA1/HLA2, MY09/MY11 and GP5 + /GP6 + primers, respectively).

### HPV genotyping

HPV-positive samples underwent genotyping in Real Time PCR using “HPV HR/LR 23 Types Detection Kit RQ” (Experteam, Italy), which identifies the 23 most common HPV types, of which 14 HR-HPVs (16, 18, 31, 33, 35, 39, 45, 51, 52, 56, 58, 59, 66, 68) e 9 LR-HPVs (6, 11, 26, 53, 67, 70, 73, 81, 82). The amplification was carried out in a 48-well plate with Step One Real Time PCR System (Applied Biosystems, Carlsbad, CA, USA), according to the manufacturer's instructions.

CT values below 37 suggested positivity to one or more viral genotypes (multiple infection).

### Total RNA extraction and HPV-RNA quantification in positive semen samples

To quantitatively detect HPV transcriptional activity in semen, we analyzed E6 and E7 expression using specific probes for the most frequent strains (HPV6, 16, 18, 31, 53, 58).

Total RNA was isolated from an aliquot of 500 µl of semen using guanidine isothiocyanate lysis buffer (Trizol, Gibco BRL, NY, USA) with a step of digestion with deoxyribonuclease I (DNase I, RNase-free, ZYMO Research, Irvine, CA, USA), according to the manufacturer’s instructions.

Reverse transcription (RT) for cDNA synthesis was carried out on 200 ng of RNA extracted from each sample in a final reaction volume of 50 μl, using the High Capacity cDNA RT kit (Invitrogen Corporation, San Diego, CA, USA), according to the manufacturer’s instructions. Quantification of mRNA was carried out using real-time 5′exonuclease RT-PCR fluorogenic assay (TaqMan PCR, Applied Biosystems) using the Light Cycler 480 II sequence detector (Roche, Monza, Italy). Specific primer pairs, at a final 600 nM concentration, and the proper probe double-labelled (6-carboxy-fluorescein [FAM] and 6-carboxy-tetramethyl-rhodamine [TAMRA], at 5′ and 3′ ends, respectively), at a final 300 nM concentration, were added to Light Cycler Probe Master Mix (Roche) in a 20 μl volume. TaqMan probes and primers (shown in Table S3) were designed to anneal in the HPV E6/E7 gene, as previously described [26]. RT-PCR conditions for amplifying target genes and GAPDH were as follows: pre-incubation 10 min at 95 °C; amplification for 45 cycles (95 °C for 10 s, 60 °C for 30 s, and 72 °C for 1 s); cooling 40 °C for 30 min. Copy numbers were calculated by means of an external standard curve generated by amplifying serial tenfold dilutions (10–10^8^ copies) of a DNA plasmid containing the E6/E7 fragment of each genotype. These type-specific standards were generated by cloning E6/E7 fragments in Topo TA vector (Invitrogen, San Diego, California, USA) [25]. The lower limit of sensitivity of the assay is about 10 copies/ng of total DNA. All samples were tested in triplicate along with positive (DNA templates purified from HPV-positive samples) and negative (cDNA from HPV-negative samples and no cDNA) controls. Glyceraldehyde-3-phosphate dehydrogenase (*GAPDH*) and β-glucuronidase (*GUS*) were used as endogenous genes for sample normalization, while protamin 1 (*PRM1*) mRNA, a sperm-specific transcript, was used as control for sperm RNA extraction, using RT-PCR (TaqMan™ Gene Expression Assay, Applied Biosystems) [27].

### Statistical analysis

Continuous variables have been expressed as a mean ± standard deviations or medians and interquartile range, where appropriate, in relation to the normality of the value distributions evaluated with the Kolmogorov–Smirnov test. Comparisons among the two groups (A and B) have been carried out using student’s *t* (independent *t* test) or Mann–Whitney *U* tests for independent samples, where appropriate. Categorical variables, expressed as percentages, are evaluated with the *χ*^2^ test. Correlations were computed using Spearman’s correlation test.

To further evaluate associations between HPV positivity and specific clinical traits, logistic regression analysis has been used and results were described as odd ratios (OR) and 95% confidence intervals (CI). A two-tailed *p*-value lower than 0.05 was considered as statistically significant. The statistical analysis was carried out using the Statistical Package for the Social Sciences (SPSS) 27.0 (SPSS Inc., Chicago, USA) software.

## Results

### Clinical data

We enrolled 185 sexually active subjects aged ≥ 18 years (36.0 ± 8.3 years, range 18–60 years). 85/185 had risk factors for HPV infection (Group A); conversely, 100/185 reported no known risk factors for viral infection (Group B). Age and BMI significantly differed between the two groups (age: 34.2 ± 9.7 vs. 37.6 ± 6.6 years, Group A vs. B respectively, *p* = 0.002; BMI: 23.8 ± 3.0 vs. 25.4 ± 3.1 kg/m^2^, respectively, *p* < 0.001).

Table 1 shows relevant demographics and medical history from the two groups. As expected, Group A had a higher incidence of genito-urinary infections (Mycoplasma spp., Chlamydia spp., Klebsiella spp., Citrobacter spp., Escherichia coli, Staphylococcus aureus, Proteus spp., Candida spp.) and other viral coinfections (HSV, HIV, HBV, HCV). On the other hand, unprotected sexual intercourses and anti-HPV vaccination rates were unsatisfactorily low in both groups (Table 1).

**Table 1:** Demographic and medical history of the two study groups

	Group A	Group B	*p*-value
Smokers	30/85 (35.3%)	31/100 (31.0%)	0.638
Alcohol (> 5 AU/week)	24/85 (28.2%)	27/100 (27.0%)	0.870
Education			
Secondary school	0/85 (0%)	9/100 (9.0%)	0.690
High school	45/85 (52.9%)	41/100 (41.0%)	
Degree/post graduate	40/85 (47.1%)	50/100 (50.0%)	
Circumcision	6/85 (7.1%)	4/100 (4.0%)	0.517
Urogenital infections	9/85 (10.6%)	0/100 (0%)	** < 0.001**
Other viral coinfections	11/85 (12.9%)	0/100 (0%)	** < 0.001**
Unprotected sexual intercourses	79/85 (92.9%)	94/100 (94.0%)	0.775
Multiple sexual partners (n°partners > 1)	43/85 (50.6%)	0/100 (0%)	** < 0.001**
Genital warts	22/85 (25.9%)	0/100 (0%)	** < 0.001**
Personal history of anogenital HPV infection	26/85 (30.6%)	0/100 (0%)	** < 0.001**
Partner history of anogenital HPV infection	35/85 (41.2%)	0/100 (0%)	** < 0.001**
Anti-HPV vaccination	13/85 (15.3%)	6/100 (6.0%)	0.051

Significant *p*-values are in bold (*χ*^2^ test)

Group A: patients with risk factors for HPV infection; Group B: patients with no risk factors for HPV infection

*AU* alcoholic unit

### Semen analysis

We detected seven azoospermic and one cryptozoospermic subject in the overall caseload; in particular, in Group A there were three azoospermic and the cryptozoospermic subject, while 4 azoospermic subjects were in Group B. These subjects were excluded from the statistical analyses of semen parameters. Table S4 describes semen parameters in the two groups.

### Verification of feasibility and detection limit of the molecular methodology

The use of the molecular standard allowed us to confirm the efficiency of the DNA extraction technique and nested PCR in identifying HPV-DNA in semen sample.

**Fig. 1:**
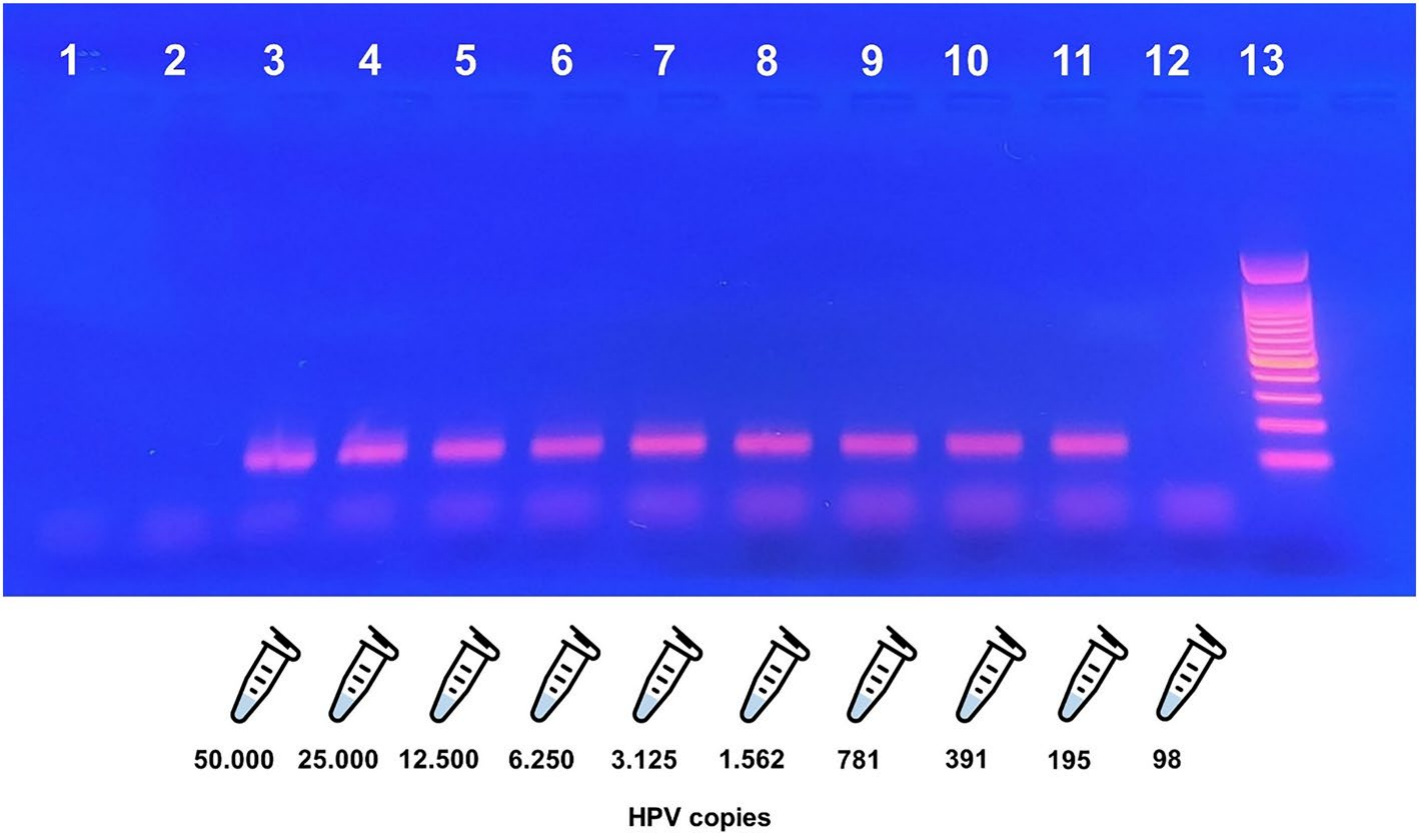
Agarose gel electrophoresis of nested PCR amplification products (size 150 bp). Lane 1: sample without DNA; lane 2: sample from a patient with negative history and physical examination for HPV infection (negative control sample); lanes 3–12: negative control sample spiked with an inactivated HPV-16 pellet (positive control sample) containing 50.000, 25.000, 12.500, 6.250, 3.125, 1.562, 781, 391, 195 and 98 HPV copies, respectively; lane 13: DNA Ladder 100 bp

We proved that nested PCR was able to reveal HPV-DNA up to a detection limit of approximately 195 copies, as shown in Fig. 1. Below this threshold it is plausible to hypothesize that the viral genome cannot be detected in semen using nested PCR as investigation technique.

### HPV-DNA detection in semen samples

**Fig. 2:**
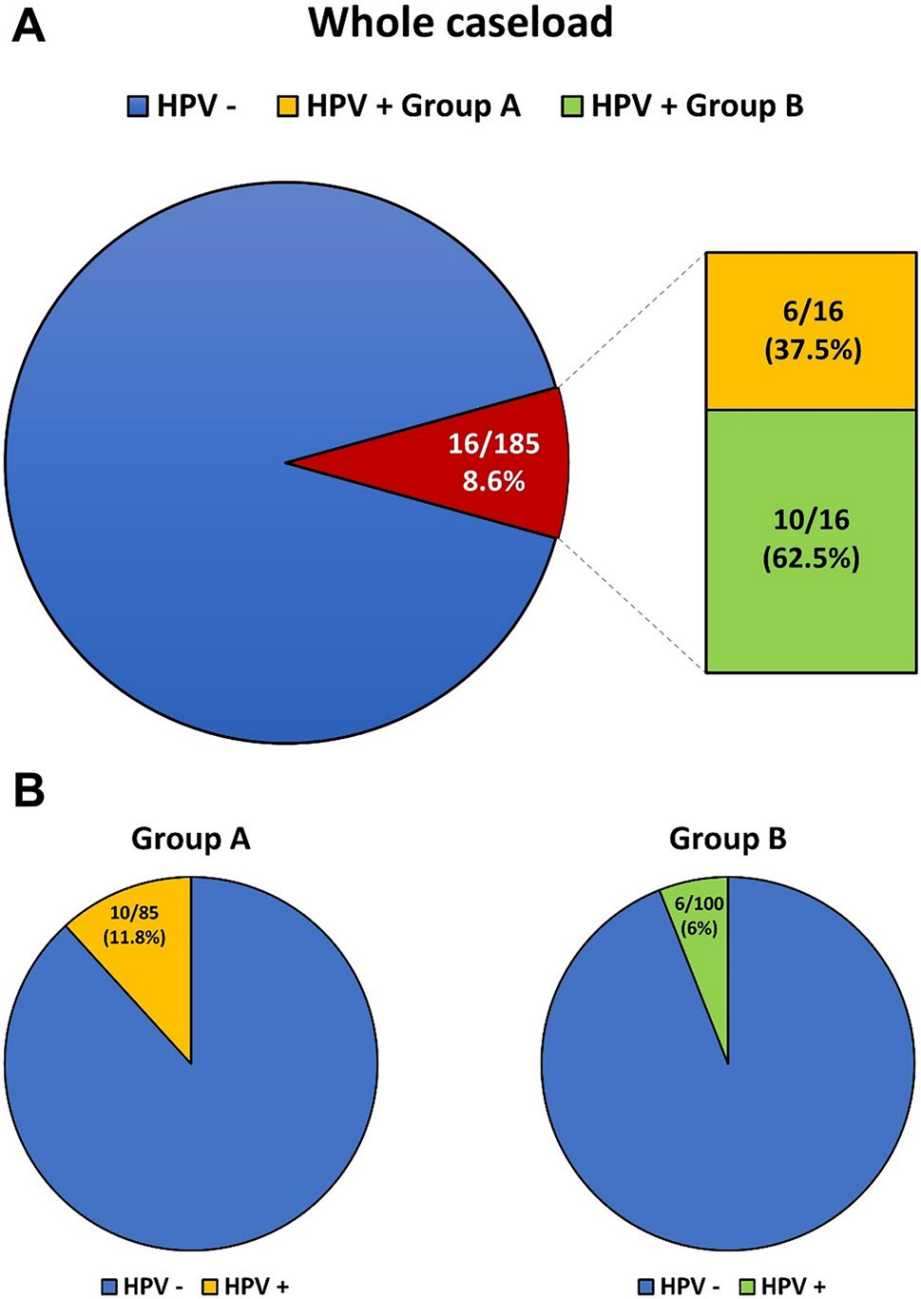
**a** Seminal HPV prevalence in the whole caseload and stratified by Risk Factors. **b** Seminal HPV prevalence in the two study groups

In the whole caseload molecular analysis revealed seminal HPV prevalence in 16/185 subjects (8.6%). Based on medical history, 10/16 (62.5%) subjects with HPV in semen had specific risk factors for HPV, while 6/16 (37.5%) reported no known risk factors for viral infection (Fig. 2a). In particular, we observed seminal HPV presence in 10/85 (11.8%) patients of Group A and in 6/100 (6%) men of Group B (*p* = 0.195) (Fig. 2b). Moreover, when considering the total of subjects with at least one partner of the couple with a HPV history (45/85 subjects), the prevalence is 11.1% (5/45 subjects), in subjects with multiple sexual partners is 11.6% (5/43 subjects), while it increases up to 27.3% when considering the subgroup of subjects with genital warts (6/22 subjects) and to 30.6% (26/85 subjects) in men with previous and/or current anogenital HPV infection (Fig. S1).

### HPV-DNA in semen and sperm parameters

Among patients with HPV-positive semen, we observed one azoospermic and one cryptozoospermic subjects (both belonging to Group A). Table 2 shows the comparison of semen parameters between HPV positive and negative patients. No significant difference in sperm parameters was found, although this may be due to the limited number of positive patients observed. Likewise, the immunological study revealed no positivity in any patient with HPV-DNA in semen.

**Table 2:** Comparison of semen parameters between HPV-positive and negative patients (median and 25th–75th percentile in brackets and significance evaluated by Mann–Whitney *U* test)

	HPV-positive patients (*n* = 14)	HPV-negative patients (*n* = 163)	*p*-value
Semen Volume (ml)	2.5 (1.9–3.8)	3.0 (2.0–4.0)	0.525
Sperm Concentration (10^6^/ml)	37.5 (12.5–65.0)	42.0 (12.0–86.0)	0.682
Total Sperm Number (10^6^/ejaculate)	98.0 (22.8–159.1)	108.0 (38.4–220.0)	0.491
Progressive Motility (%)	40.0 (20.0–55.0)	45.0 (15.0–50.0)	0.560
Abnormal Forms (%)	90.0 (86.0–94.0)	90.0 (88.0–96.0)	0.463
Leukocytes (10^6^/ml)	0.6 (0.3–1.1)	0.7 (0.4–0.9)	0.624
Sperm Viability (%)	65.0 (52.0–82.0)	67.0 (50.5–75.0)	0.659

Crypto/Azoospermic subjects have been excluded from analyses

Finally, we detected that the only variables significantly associated with the risk of HPV positivity in seminal fluid were the presence of genital warts (OR 5.79, 95% CI 1.84–18.23, *p* = 0.003) and previous anogenital HPV infection (OR 4.93, 95% CI 1.62–14.98, *p* = 0.005). The other relevant clinical risk factor investigated, multiple sexual partners, was found not to be significantly associated with HPV-DNA presence in semen (OR 1.51, 95% CI 0.50–4.62, *p* = 0.466). Regarding semen cytological parameters, none was found to be significantly associated with the presence of HPV in semen.

### HPV genotypes

Genotyping of HPV-positive semen samples allowed to detect both LR-HPVs and HR-HPVs, as shown in Table 3. Only for one patient tested positive for the qualitative research of viral DNA by nested PCR it was not possible to identify the specific genotype, suggesting the presence of a strain not detectable by the kit used in our study. Similarly, it should be stressed that for each sample it is not possible to exclude positivity to other non-investigated genotypes. In samples in whom genotyping was successful, 9/15 men (60%) showed a multiple infection and 13/15 (86.7%) showed positivity to HR-HPVs.

**Table 3:** Genotyping of HPV-positive samples

	Patient Group	LR genotype	HR genotype
Patient #9	Group A	6	nd
Patient #10	Group A	6	nd
Patient #20	Group A	nd	58, 66
Patient #61	Group A	nd	33, 51
Patient #71	Group A	nd	31, 66
Patient #109	Group A	nd	52, 58
Patient #166	Group A	nd	nd
Patient #175	Group A	81	18, 35, 56, 59
Patient #178	Group A	nd	45
Patient #185	Group A	nd	16
Patient #36	Group B	53	56
Patient #73	Group B	nd	66
Patient #74	Group B	nd	16, 31, 58
Patient #112	Group B	nd	56
Patient #126	Group B	26, 53, 67	33
Patient #157	Group B	73	39

### HPV-RNA expression in positive semen samples

To ascertain whether HPV-DNA positivity were also associated to HPV genome transcriptional activity, the presence of mRNA copies of the more common low-risk (HPV6, 11) and high-risk (16, 18, 31, 33, 53, 58) genotypes was tested with sensitive Real Time PCR assays (RT-PCR), after DNA removal. However, based on the biological material available and the genotype-specific HPV probes used, 6 out of 16 samples with HPV-DNA in semen could be analyzed for E6 and E7 HPV-RNA expression (Table S5).

**Fig. 3:**
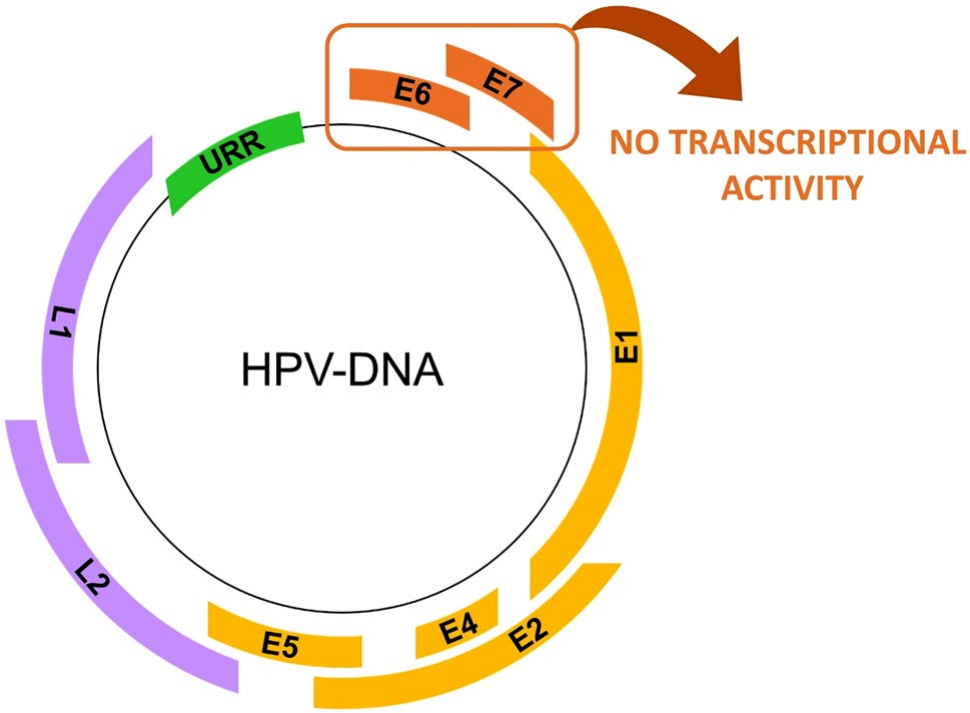
Schematic representation of the HPV genome and E6/E7 expression in positive semen samples analyzed by Real Time PCR. URR: Upstream Regulatory Region; E1, E2, E4, E5, E6, E7: early genes; L1, L2: late genes

While detecting the expression of the endogenous genes (*GAPDH* and *GUS*) and confirming an efficient sperm RNA extraction (*PRM1* CT mean = 29.01), RT-PCR did not detect HPV-E6 and E7 expression in any HPV-DNA positive semen sample tested (Fig. 3), suggesting the absence of potentially infectious virions in this biological fluid.

### Sperm-Mar test

The immunological study performed in our caseload highlighted the positivity to ASAs only in three semen samples, two of which belonging to Group A and one to Group B. The ASAs binding percentages were the following:

- Patient #39 (Group B): 95% positive to IgG class with binding on the head and tail; 20% positive to IgA class with binding exclusively on the tail;
- Patient #116 (Group A): 90% positive to IgG class with binding on the tail and 20% to IgA class exclusively tail;
- Patient #160 (Group A): 20% positive to IgG with binding on the tail and negative to IgA.

These samples were negative for HPV presence in semen investigated by nested PCR.

## Discussion

### HPV in semen samples

HPVs are the etiological agents of one of the most common sexually transmitted diseases causing a variety of clinical manifestations ranging from warts to cancer.

In human semen HPV shows a variable prevalence from 1.3% to 72.9% with a peak of 65.4% in reproductive age [28]. Unlike women in whom the prevalence is high after the onset of sexual activity and then shows a decrease, in men the prevalence of infection remains high even in older age [29].

In the male genital tract, HPV can be localized in different anatomic sites, such as penis shaft, glans, coronal sulcus, prepuce, scrotum, anal and perianal canal but also urethra and semen [30]. Depending on the anatomic site, HPV prevalence and load display a significant variability showing higher proportions in samples obtained by epithelial scraping of external genitalia than in semen [31, 32]. In the latter site, the literature shows an extremely variable prevalence of HPV, ranging between 7.8% [33] and 53.8% [13].

To date, HPV detection in semen is not much applied in clinical practice and no protocol is recognized as gold standard for its detection in this biological fluid. Literature shows different methodologies to detect HPV in semen, such as PCR, Real Time PCR and hybridization assays. As each of these techniques exhibits a different ability to detect the virus based on the specific molecular principle, HPV detectability in semen might change. This could explain at least in part the wide range of seminal HPV prevalence reported in literature (Table 4). However, it should be stressed that all the above-mentioned methods are valid to detect HPV-DNA but using the same technique the prevalence may differ, suggesting the contribution of several factors that are often not easily identifiable. Unfortunately, to date there are no studies aimed at comparing the ability of the various techniques to detect HPV in semen. Thus, the identification of a gold standard protocol is lacking in literature. In 2009 Coutlee et al. compared several methods for detection and typing of HPV in biological fluids, although semen was not included [34]. Describing benefits and limitations of the various molecular procedures, this study showed that nucleic acid amplification techniques are ideal methods to detect infection with low viral burden. Conversely, other molecular strategies, such as in situ hybridization assays, could cause false negative results in presence of lower amounts of HPV-DNA.

**Table 4:** Prevalence of seminal HPV presence in literature investigated

Reference	*N* patients	Country	HPV prevalence in semen
(a) With PCR/nested PCR			
[34]	216 infertile men	Japan	12.5%
[35]	31 fertile men	Finland	19.4%
[16]	96 infertile men	Iran	17.4%
[8]	729 infertile men	Europe	15.5%
[36]	25 infertile men	Brazil	28%
[37]	90 men: 50 oligo 20 azoo 20 normo	Iran	30% in oligo 40% in azoo 15% in normo
[12]	200 volunteers: 100 with previous sexual intercourse 100 without previous sexual intercourse	Italy	10% in men with previous sexual intercourse 0% in men without previous sexual intercourse
[13]	290 men: 26 with genital warts 66 with HPV + partners 108 infertile patients 90 fertile controls	Italy	53.8% in men with genital warts 40.9% in men with HPV + partners 10.2% in infertile men 2.2% in fertile men
[38]	24 infertile men	China	25% for HPV-16 DNA 46% for HPV-18 DNA
Present study	185 men: 85 with risk factors (Group A) 100 with no known risk factors for HPV infection (Group B)	Italy	Whole caseload: 8.6% Group stratification: 11.8% (10/85) in Group A 6.0% (6/100) in Group B
(b) With Real Time PCR			
[39]	100 infertile men	Italy	38%: 20% positive for LR-HPV 18% positive for HR-LR
[7]	161 infertile men	Belgium	14.8% per IUI cycle
[40]	425 men: 97 sperm donors 328 infertile men	Czech Republic	6.2% for HR-HPV and 1.03% for LR-HPV in sperm donors 11.9% for HR-HPV and 4.88% for LR-HPV in infertile men
[41]	100 infertile men	Lithuania	20%
[10]	140 men: 70 infertile 70 fertile	Iran	11.43% in infertile men 0% in fertile men
(c) With hybridization assays			
[42]	117 men of couples with idiopathic recurrent pregnancy loss	Italy	20%
[43]	117 infertile partners of HPV-positive women	Italy	40.2%
[9]	22 partners of HPV + women with HSIL	Italy	45%
[44]	38 men: 22 infertile 9 fertile 7 fertile with genital warts	Mexico	27% in infertile men 0% in fertile men 71% in men with genital condyloma
[45]	213 volunteers: 33 with flat penile lesions 5 with condyloma	Netherlands	27.2%
[31]	340 infertile men	Slovenia	13.61%
[46]	76 donors	Denmark	26%
[32]	308 infertile men	Italy	7.8%
[11]	1138 men: 615 infertile 523 fertile	China	12.48% in the whole caseload 17.4% in infertile men 6.7% in fertile men
[47]	188 donors	Denmark	16%

For this reason, one of the main purposes of our study was to evaluate the effective ability of a common molecular biology technique, the nested PCR, to identify HPV genome in seminal samples. Contamination of a negative control with an inactivated HPV-16 pellet allowed us to monitor the efficiency of the molecular methods used in our study, confirming the ability of the nested PCR to reveal HPV-DNA in semen up to a detection limit of approximately 195 copies. Below this threshold it is reasonable to hypothesize that this technique could lead to false negatives in samples with a reduced viral load.

In our study, the data about seminal HPV prevalence from the two groups resulted lower than that shown by most papers in previous literature [7–13, 16, 32, 35–48] (Table 4). The low prevalence we found would not seem to be due to vaccination, which resulted inadequate in the overall caseload (10.3%, 19/185 patients). This discrepancy may derive from multiple factors, including geographical area from which the recruited subjects come, molecular methods chosen to reveal the viral genome, numerosity of the caseload and criteria by which the patients were selected. In particular, literature data show a HPV prevalence ranging from 4.88 to 46% in semen of infertile patients [7, 8, 10, 11, 13, 16, 32, 33, 35, 37–43, 45], and from 10 to 71% in semen of men with risk factors for viral infection [9, 12, 13, 44–46]. The prevalence we observed in semen samples of Group A (11.8%) is within the lower range shown by the literature for subjects with risk factors for HPV infection.

As expected, considering the total number of HPV-DNA positive samples detected in our study, the Group A displayed a higher HPV prevalence in semen compared to the Group B (62.5% vs. 37.5%, respectively). It should be stressed that the positivity to HPV in semen of patients with neither risk factors nor visible genital lesions suggests the presence of an unrecognized infection with a potential negative impact on reproductive health.

In Group A, 10.6% and 12.9% of patients were affected by urogenital and viral coinfections, respectively (compared to none in Group B), which further increase the probability of HPV infection. This observation agreed with the study of La Vignera et al. (2015), who demonstrated that patients with male accessory gland infections showed a significantly higher frequency of HPV infection compared with fertile controls [49].

Our results highlighted that the presence of HPV-DNA in semen was strongly associated with certain risk factors, such as sexual intercourse with multiple partners, the presence of genital warts and a history of previous HPV infection. Specifically, the risk of detecting the viral genome in semen was increased by approximately sixfold in the presence of genital warts and fivefold in the presence of previous infection.

To our knowledge, this is one of the few studies currently in the literature aiming to evaluate any association between HPV in semen and the presence of risk factors. In particular, the relatively high prevalence of HPV in the semen of men with genital warts (27.3%) and previous and/or current anogenital infection (30.6%) suggests the importance of HPV semen screening in this category of patients. In agreement with our data, also Foresta et al. reported a high HPV prevalence in semen of men with genital warts and men with HPV-positive female partners (53.8% vs. 40.9%, respectively) [13]. Subsequently, analyzing HPV prevalence in semen of 213 healthy male volunteers of which 15% with flat penile lesions and 2% with condyloma acuminata, Luttmer et al. proved that HPV-DNA in semen was associated with HPV infections of the penile epithelium. Moreover, as well as in our caseload, not all clinically detected flat penile lesions were correlated with HPV detection in semen [46]. Afterwards Cortés-Gutiérrez et al. found a HPV prevalence of 71% in semen of fertile men displaying genital warts [45]. Finally, in 2019 Capra et al. investigated HPV-DNA in semen of 22 men with female HPV-positive partner affected by high-grade squamous intraepithelial lesions (HSIL), finding seminal presence of HPV in 45% of cases. Interestingly, none of the infected males showed visible lesions [25]. As observed by Capra et al., also in our study not all HPV-positive patients exhibited visible genital lesions typical of viral infection.

### HPV-DNA in semen and sperm parameters

The impact of seminal HPV presence on sperm parameters is a controversial topic. Most studies have observed a reduction of semen quality in HPV-positive men and the parameter most impaired seems to be sperm motility [7, 8, 10–12, 16, 35, 38, 39, 42], the reduction of which is intrinsic in the infertile nature of the subjects studied.

Some authors have also revealed the presence of ASAs, suggesting that HPV in semen may represent an antigenic stimulation that would contribute to further reduce male fertility [15, 16]. However, other studies have found no significant association between seminal HPV presence and low semen quality [32, 33, 37, 43], not even in relation to sperm chromatin integrity [43, 45, 47]. Recently, Cannarella et al. found no significant difference in sperm concentration, total sperm count, progressive motility, morphology and leukocyte concentration between LR-HPV positive patients and controls with no evidence of HPV-DNA in semen, despite the prevalence of oligozoospermia and leukocytospermia were significantly higher in LR-HPV positive men [40]. Moreover, in another recent study, HPV in semen did not appear to correlate with any sperm parameters, excluding progressive motility and morphology; HPV positivity did not even modify DFI rates, except when comparing HR and LR genotypes, suggesting that HR-HPVs could specifically affect sperm DNA integrity [44].

In agreement with these latest studies, we found that the presence of HPV in semen was not correlated with impaired semen quality. However, it should be stressed that the lack of correlation may be due to the small number of positive patients found (16/185). Moreover, unlike the few previous studies [15, 16], none of subjects with HPV in semen showed ASAs.

Likewise, we did not observe associations between sperm parameters and the risk of detecting HPV in semen. As this may have been influenced by the relatively low percentage of patients with HPV-positive semen samples, we cannot rule out whether inflammatory action of HPV infection in the male urogenital tract may exert a potential impact on semen quality.

### HPV genotyping

In patients in whom genotyping was successful, we observed the presence of strains considered to be at high risk for the development of cervical cancer in 13/15 subjects (86.7%), indicating the need for a careful gynecological monitoring of women with HPV-positive partner.

The genotypes identified in our study are analogous to those found in most of the previous literature [7–9, 32, 36, 38, 45, 47] but different from those discovered by other authors [10–13, 33, 42], suggesting a different prevalence based on the geographic area examined. It is not possible to exclude the variability deriving also from the different molecular investigations used to detect and to genotype the viral genome. Furthermore, it should be stressed that the low concentrations in which HPV could be present in a biological fluid such as semen, may underestimate the study of its prevalence and the assessment of the various strains.

### Clinical impact of seminal HPV presence

In recent years, attention regarding the potential impact of HPV infection on andrological health has increased and several papers have advanced the hypothesis that HPV may constitute a risk factor for male infertility [10, 11, 13, 38] and could impact on both natural and assisted reproductive outcomes, such as pregnancy rate and miscarriage rate.

Although some authors demonstrated the ability of HPV to bind the head of spermatozoa at the equatorial segment [12, 14, 35, 46, 48, 50], it remains to be investigated the origin of HPV-DNA in seminal fluid and its viral activity. Luttmer et al. reported a firm association between HPV in semen and penile scrapes, suggesting that the presence of the viral genome in semen may be due to contamination of HPV-positive exfoliated keratinocytes from the penile epithelium [46]. To corroborate this hypothesis, the authors performed fluorescence in situ hybridization (FISH) for HPV-DNA and immunocytochemistry for the HPV-L1 and HPV-E4 proteins, proving the presence of viral genome on spermatozoa in agreement with previous studies [12, 14, 48, 50], but no HPV-L1. This observation would demonstrate that HPV positivity in semen could be caused by free viral DNA that is released from exfoliated keratinocytes and that adheres to sperm heads [46].

In line with this advice, a recent study revealed HPV-DNA also in semen of infertile patients affected by azoospermia with an infection rate of 14.3%, suggesting the hypothesis of a contamination from penile or urethral epithelial cells as a possible origin of HPV-DNA found in semen [35].

The detection of seminal HPV in azoospermic men was observed also in a previous study with a prevalence of 40% in this subgroup of patients [38].

In our study, we detected HPV-DNA in seminal plasma of a patient affected by cryptozoospermia (patient #20) and one with azoospermia (patient #175), suggesting that the presence of HPV in semen would not necessarily be linked to the presence of spermatozoa but could result from a contamination due to epithelial desquamation of the genital tract, despite the absence of visible genital lesions.

In support of this hypothesis, we found no potentially infectious traces of HPV in semen as demonstrated by the absence of viral RNA transcripts in positive samples. Our study is one of the few that has investigated HPV expression in human semen. Only Lai et al. detected HPV16 and 18 RNA in seminal fluid suggesting that semen may act as vector for the transmission of HPV [51]. However, further studies will be needed to assess the infectiousness of seminal HPV and its impact on reproductive health.

It should be highlighted that, whatever the origin of HPV, its detection in semen must be an alarm for clinicians because it suggests that in the male genital tract the virus is present and could be carried to uterine cervix of female partner with negative consequences not only for her health but also in both natural and assisted reproduction. To date, the impact of HPV-DNA, although not transcriptionally active, on fertilization and embryonic development is not yet clear but a putative negative effect on reproductive outcomes, especially in ART, cannot be excluded, as suggested by previous literature [17–20].

In 2011, Foresta et al. demonstrated that HPV16-transfected human spermatozoa were able to penetrate the hamster oocyte and viral genes were actively transcribed by the penetrated oocyte [14]. To date, the possible impact of HPV on embryonic development is not well defined but in vitro experiments proved that HPV-transfected trophoblast cells show an increased rate of apoptosis and a reduced placental invasion into the uterine wall [52–54]. This evidence could explain the increased risk of miscarriage and a reduced chance of ongoing pregnancy in patients undergoing ART, as reported by a recent meta-analysis that elucidated the effects of seminal HPV presence on reproductive outcomes [55]. In addition, a recent study showed a significant association between the presence of HR-HPV DNA in semen and recurrent pregnancy loss, suggesting an alleged detrimental impact of the seminal HPV presence on reproductive success [43]. Finally, it should be stressed that the possible consequences of HPV on ART treatment outcomes could represent a further burden with a negative impact on emotional, psychological and sexual aspects of the couple undergoing ART programs [56], requiring a dedicated evaluation.

## Conclusions

HPV is one of the major sexually transmitted viruses that can also be found in semen but currently its detection in this biological fluid is not much applied in clinical practice. However, searching for seminal HPV presence may play a pivotal role in the andrological work-up for preconceptional screening because this virus could affect male fertility and semen can act as a vehicle for horizontally and vertically transmission impacting on reproductive health.

The higher HPV prevalence in semen of men with risk factor for infection points out the importance of screening in both partners of a couple for the purpose of natural fertilization, but also for ART where the risk of injecting sperm containing HPV-DNA is possible. Nevertheless, further studies will be needed to estimate the infectiousness of HPV in semen and its impact on reproductive outcomes.

In the light of the obtained results, we can conclude that our study shows strengths but also some limitations. First, we proved the ability of nested PCR to reveal HPV-DNA in semen up to a detection limit of approximately 195 copies. Moreover, the lack of HPV-E6 and E7 expression in HPV-DNA positive semen sample tested suggests the absence of potentially infectious virions in this biological fluid. Nevertheless, this result must still represent a clinical alarm as, to date, although not transcriptionally active, the effect of HPV-DNA on reproductive outcomes is not yet clear. However, some limitations of the study are related to the technical restrictions of the methods used for HPV detection and genotyping. In particular, we cannot exclude false negative results due to the difficulty of detecting HPV-DNA by nested PCR in seminal samples with viral load below the aforementioned detection limit. Likewise, positivity to genotypes not detectable by the kit used in this study cannot be ruled out. These limitations would lead to an underestimation of the HPV prevalence in semen of the enrolled patients. Furthermore, the results obtained will have to be confirmed in a wider cohort to corroborate the clinical importance of seminal HPV presence.

### Supplementary Information

Below is the link to the electronic supplementary material.

Supplementary file1 (DOCX 172 KB)

Supplementary file2 (DOCX 16 KB)

Supplementary file3 (DOCX 14 KB)

Supplementary file4 (DOCX 18 KB)

Supplementary file5 (DOCX 18 KB)

Supplementary file6 (DOCX 17 KB)

## Data Availability

No data or material to share.
