# Supplementary figures and images for: Molecular study of the presence and transcriptional activity of HPV in semen
Source: J Endocrinol Invest. 2023 Aug 16;47(3):557–70. doi: 10.1007/s40618-023-02167-4 (PMC10904563; doi:10.1007/s40618-023-02167-4)

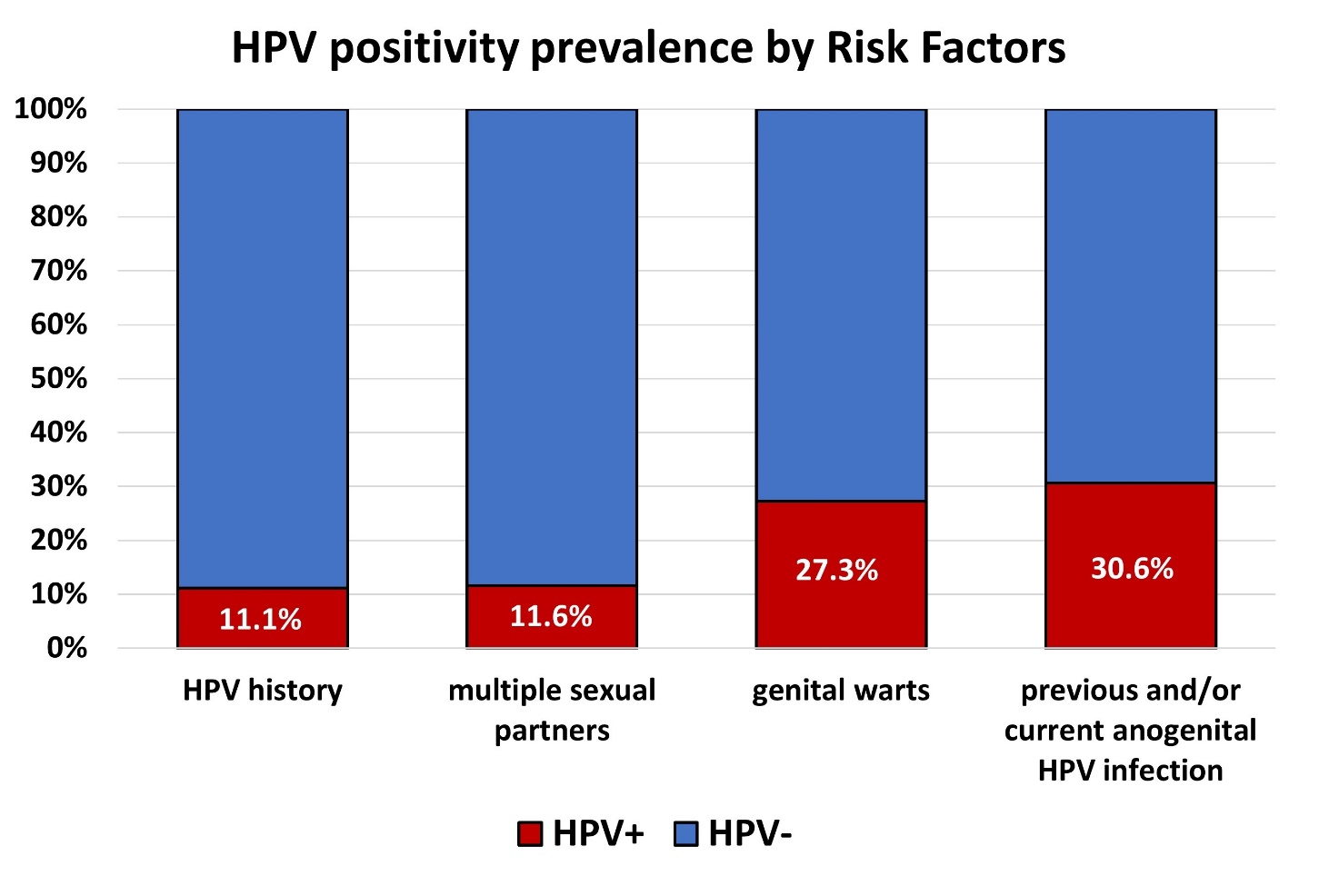

Supplement: Supplementary file 1 — Supplementary file1 (DOCX 172 KB) [file 40618_2023_2167_MOESM1_ESM.docx]
