# Supplementary material for: Molecular study of the presence and transcriptional activity of HPV in semen
Source: J Endocrinol Invest. 2023 Aug 16;47(3):557–70. doi: 10.1007/s40618-023-02167-4 (PMC10904563; doi:10.1007/s40618-023-02167-4)
Supplement: Supplementary file 2 — Supplementary file2 (DOCX 16 KB) [file 40618_2023_2167_MOESM2_ESM.docx]

**Article Title:** “Molecular study of the presence and transcriptional activity of HPV in semen”

**Journal name:** Journal of Endocrinological Investigation

**Authors’ names:** Fabiana Faja^1^ · Francesco Pallotti^1^ · Serena Bianchini^1^ · Alessandra Buonacquisto^1^ · Gaia Cicolani^1^ · Anna Chiara Conflitti^1^ · Matteo Fracella^2^ · Eugenio Nelson Cavallari^2^ · Francesca Sciarra^3^ · Alessandra Pierangeli^2^ · Donatella Paoli^1^ · Andrea Lenzi^1^ · Guido Antonelli^2^ · Francesco Lombardo^1^ · Daniele Gianfrilli^3^

**Affiliations:**

^1^ Laboratory of Seminology - “Loredana Gandini” Sperm Bank, Department of Experimental Medicine, “Sapienza” University of Rome, 00161 Rome, Italy

^2^ Laboratory of Microbiology and Virology, Department of Molecular Medicine, “Sapienza” University of Rome, 00185 Rome, Italy

^3^ Section of Medical Pathophysiology and Endocrinology, Department of Experimental Medicine, “Sapienza” University of Rome, 00161 Rome, Italy

**E-mail address of the corresponding author:** donatella.paoli@uniroma1.it

**Table S1** List of Risk Factors used to stratify the caseload

| **Risk Factors** |
| --- |
| Urogenital infections (such as Mycoplasma spp., Chlamydia spp., Klebsiella spp., Citrobacter spp., Escherichia coli, Staphylococcus aureus, Proteus spp., Candida spp.) |
| Other viral coinfections (such as HSV, HIV, HBV, HCV) |
| Unprotected sexual intercourses |
| Multiple sexual partners (n°partners > 1) |
| Genital warts |
| Personal history of anogenital HPV infection |
| Partner history of anogenital HPV infection diagnosed within 12 months |
| Anti-HPV vaccination |
