# Supplementary material for: Molecular study of the presence and transcriptional activity of HPV in semen
Source: J Endocrinol Invest. 2023 Aug 16;47(3):557–70. doi: 10.1007/s40618-023-02167-4 (PMC10904563; doi:10.1007/s40618-023-02167-4)
Supplement: Supplementary file 3 — Supplementary file3 (DOCX 14 KB) [file 40618_2023_2167_MOESM3_ESM.docx]

**Article Title:** “Molecular study of the presence and transcriptional activity of HPV in semen”

**Journal name:** Journal of Endocrinological Investigation

**Authors’ names:** Fabiana Faja^1^ · Francesco Pallotti^1^ · Serena Bianchini^1^ · Alessandra Buonacquisto^1^ · Gaia Cicolani^1^ · Anna Chiara Conflitti^1^ · Matteo Fracella^2^ · Eugenio Nelson Cavallari^2^ · Francesca Sciarra^3^ · Alessandra Pierangeli^2^ · Donatella Paoli^1^ · Andrea Lenzi^1^ · Guido Antonelli^2^ · Francesco Lombardo^1^ · Daniele Gianfrilli^3^

**Affiliations:**

^1^ Laboratory of Seminology - “Loredana Gandini” Sperm Bank, Department of Experimental Medicine, “Sapienza” University of Rome, 00161 Rome, Italy

^2^ Laboratory of Microbiology and Virology, Department of Molecular Medicine, “Sapienza” University of Rome, 00185 Rome, Italy

^3^ Section of Medical Pathophysiology and Endocrinology, Department of Experimental Medicine, “Sapienza” University of Rome, 00161 Rome, Italy

**E-mail address of the corresponding author:** donatella.paoli@uniroma1.it

**Table S2** Primers used to test the presence of amplifiable DNA (HLA1/HLA2) and to investigate the presence of HPV in semen through nested PCR (MY09/MY11 and GP5+/GP6+)

| **Primers** | **Sequences** |
| --- | --- |
| HLA1 | 5’-GTC CTG CAG GTG TAA ACT TGT ACC AG-3’ |
| HLA2 | 5’-CAC GGA TCC GGT AGC AGC GGA GAG TTG-3’ |
| MY09 | 5’-CGT CCM ARR GGA WAC TGA TC-3’ |
| MY11 | 5’-GCM CAG GGW CAT AAY AAT GG-3’ |
| GP5+ | 5’-TTT GTT ACT GTG GTA GAT ACT AC-3’ |
| GP6+ | 5’-GAA AAA TAA ACT GTA AAT CAT ATT C-3’ |
