# Supplementary material for: Molecular study of the presence and transcriptional activity of HPV in semen
Source: J Endocrinol Invest. 2023 Aug 16;47(3):557–70. doi: 10.1007/s40618-023-02167-4 (PMC10904563; doi:10.1007/s40618-023-02167-4)
Supplement: Supplementary file 4 — Supplementary file4 (DOCX 18 KB) [file 40618_2023_2167_MOESM4_ESM.docx]

**Article Title:** “Molecular study of the presence and transcriptional activity of HPV in semen”

**Journal name:** Journal of Endocrinological Investigation

**Authors’ names:** Fabiana Faja^1^ · Francesco Pallotti^1^ · Serena Bianchini^1^ · Alessandra Buonacquisto^1^ · Gaia Cicolani^1^ · Anna Chiara Conflitti^1^ · Matteo Fracella^2^ · Eugenio Nelson Cavallari^2^ · Francesca Sciarra^3^ · Alessandra Pierangeli^2^ · Donatella Paoli^1^ · Andrea Lenzi^1^ · Guido Antonelli^2^ · Francesco Lombardo^1^ · Daniele Gianfrilli^3^

**Affiliations:**

^1^ Laboratory of Seminology - “Loredana Gandini” Sperm Bank, Department of Experimental Medicine, “Sapienza” University of Rome, 00161 Rome, Italy

^2^ Laboratory of Microbiology and Virology, Department of Molecular Medicine, “Sapienza” University of Rome, 00185 Rome, Italy

^3^ Section of Medical Pathophysiology and Endocrinology, Department of Experimental Medicine, “Sapienza” University of Rome, 00161 Rome, Italy

**E-mail address of the corresponding author:** donatella.paoli@uniroma1.it

**Table S3** Primers and probes sequences used to analyze E6 and E7 expression of the specific HPV genotypes detected in positive semen samples

| **Target** | **Primers and probes sequences** |
| --- | --- |
| HPV6 | **F**: 5'-CAA GGC ACG GTT CAT AAA GCT A-3'  **R**: 5'-TAG GGT AAC ATG TCT TCC ATG CAT-3'  **Probe**: 5'-FAM-TAC GTG GAA GGG TCG CTG CCT ACA CT-TAMRA-3' |
| HPV16 | **F**: 5’-GAA CTG CAA TGT TTC AGG ACC C-3’  **R**: 5’-TGT ATA GTT GTT TGC AGC TCT GTG C-3’  **Probe**: 5’-FAM-CAG GAG CGA CCC AGA AAG TTA CCA CAT TTA-TAMRA-3’ |
| HPV18 | **F**: 5’-GGA ACT TAC AGA GGT ATT TGA ATT TGC-3’  **R**: 5’-TGT CTC CAT ACA CAG AGT CTG AAT AAT G-3’  **Probe**: 5’-FAM-CCG CAT GCT GCA TGC CAT AAA TGT-TAMRA-3’ |
| HPV31 | **F**: 5'-CCT ACG TTG CAA GAC TAT GTG TTA GAT-3'  **R**: 5'-CTG AGC TGT CGG GTA ATT GCT-3'  **Probe**: 5'-FAM- CAA CCT GAG GCA ACT GAC CTC CAC T-TAMRA-3’ |
| HPV53 | **F**: 5'-CCA CGT ACA TTG CAC CAG CTA-3'  **R**: 5'-TTG CAG AAC ACA CAG CCA AGT-3'  **Probe**: 5'-FAM-AAG TTG TGA ATA AAC CAT TGC TGG AGC TGC-TAMRA-3' |
| HPV58 | **F**: 5'-ACG GAC ATT GCA TGA TTT GTG T-3'  **R**: 5'-TGC AAA GTC TTT TTG CAT TCA AC-3'  **Probe**: 5'-FAM-TGG AGA CAT CTG TGC ATG AAA TCG AAT TG-TAMRA-3' |
| GAPDH | **F:** 5′-CAT GGG TGT GAA CCA TGA GAA-3′  **R**: 5′-GGT CAT GAG TCC TTC CAC GAT-3′  **Probe**: 5′-FAM-AAC AGC CTC AAG ATC ATC AGC AAT GCC T-TAMRA-3′ |
| GUS | **F:** 5′-TCTGTCAAGGGCAGTAACCTG-3′  **R:** 5′-GCCCACGACTTTGTTTTCTG-3′  **Probe:** 5’-FAM-TATGTCTTTCGATATGCAGCCAAGTTTTACCG-TAMRA-3’ |
