# Supplementary material for: Molecular study of the presence and transcriptional activity of HPV in semen
Source: J Endocrinol Invest. 2023 Aug 16;47(3):557–70. doi: 10.1007/s40618-023-02167-4 (PMC10904563; doi:10.1007/s40618-023-02167-4)
Supplement: Supplementary file 5 — Supplementary file5 (DOCX 18 KB) [file 40618_2023_2167_MOESM5_ESM.docx]

**Article Title:** “Molecular study of the presence and transcriptional activity of HPV in semen”

**Journal name:** Journal of Endocrinological Investigation

**Authors’ names:** Fabiana Faja^1^ · Francesco Pallotti^1^ · Serena Bianchini^1^ · Alessandra Buonacquisto^1^ · Gaia Cicolani^1^ · Anna Chiara Conflitti^1^ · Matteo Fracella^2^ · Eugenio Nelson Cavallari^2^ · Francesca Sciarra^3^ · Alessandra Pierangeli^2^ · Donatella Paoli^1^ · Andrea Lenzi^1^ · Guido Antonelli^2^ · Francesco Lombardo^1^ · Daniele Gianfrilli^3^

**Affiliations:**

^1^ Laboratory of Seminology - “Loredana Gandini” Sperm Bank, Department of Experimental Medicine, “Sapienza” University of Rome, 00161 Rome, Italy

^2^ Laboratory of Microbiology and Virology, Department of Molecular Medicine, “Sapienza” University of Rome, 00185 Rome, Italy

^3^ Section of Medical Pathophysiology and Endocrinology, Department of Experimental Medicine, “Sapienza” University of Rome, 00161 Rome, Italy

**E-mail address of the corresponding author:** donatella.paoli@uniroma1.it

**Table S4** Sperm parameters (mean ± standard deviation and median in brackets) of the two study groups. Group A: patients with risk factors for HPV infection; Group B: patients with no risk factors for HPV infection. Crypto/Azoospermic subjects have been excluded from analyses

|  | Group A  (n = 81) | Group B  (n = 96) |
| --- | --- | --- |
| Semen Volume (ml) | 3.0 ± 1.6  (2.6) | 3.2 ± 1.7  (3.0) |
| Sperm Concentration (10^6^/ml) | 61.0 ± 46.5  (55.0) | 44.2 ± 44.7  (28.0) |
| Total Sperm Number (10^6^/ejaculate) | 171.4 ± 143.7  (150.0) | 126.1 ± 139.8  (80.0) |
| Progressive Motility (%) | 39.6 ± 18.8  (50.0) | 30.5 ± 19.2  (30.0) |
| Abnormal Forms (%) | 88.5 ± 14.4  (90.0) | 90.0 ± 16.5  (92.0) |
| Leukocytes (10^6^/ml) | 0.8 ± 0.6  (0.7) | 0.8 ± 0.6  (0.6) |
| Sperm Viability (%) | 69.4 ± 13.3  (71.0) | 56.9 ± 18.4  (61.5) |
