# Supplementary material for: Molecular study of the presence and transcriptional activity of HPV in semen
Source: J Endocrinol Invest. 2023 Aug 16;47(3):557–70. doi: 10.1007/s40618-023-02167-4 (PMC10904563; doi:10.1007/s40618-023-02167-4)
Supplement: Supplementary file 6 — Supplementary file6 (DOCX 17 KB) [file 40618_2023_2167_MOESM6_ESM.docx]

**Article Title:** “Molecular study of the presence and transcriptional activity of HPV in semen”

**Journal name:** Journal of Endocrinological Investigation

**Authors’ names:** Fabiana Faja^1^ · Francesco Pallotti^1^ · Serena Bianchini^1^ · Alessandra Buonacquisto^1^ · Gaia Cicolani^1^ · Anna Chiara Conflitti^1^ · Matteo Fracella^2^ · Eugenio Nelson Cavallari^2^ · Francesca Sciarra^3^ · Alessandra Pierangeli^2^ · Donatella Paoli^1^ · Andrea Lenzi^1^ · Guido Antonelli^2^ · Francesco Lombardo^1^ · Daniele Gianfrilli^3^

**Affiliations:**

^1^ Laboratory of Seminology - “Loredana Gandini” Sperm Bank, Department of Experimental Medicine, “Sapienza” University of Rome, 00161 Rome, Italy

^2^ Laboratory of Microbiology and Virology, Department of Molecular Medicine, “Sapienza” University of Rome, 00185 Rome, Italy

^3^ Section of Medical Pathophysiology and Endocrinology, Department of Experimental Medicine, “Sapienza” University of Rome, 00161 Rome, Italy

**E-mail address of the corresponding author:** donatella.paoli@uniroma1.it

**Table S5** HPV-RNA expression and sperm parameters of HPV-DNA positive semen samples. Sperm parameters were not reported for patients #20 and #175 as these samples resulted cryptozoospermic and azoospermic, respectively. ND: not detectable

| **Patient** | **Group** | **Probes** | **E6/E7**  **RNA expression** | **Sperm Concentration**  **(10^6^/ml)** | **Total Sperm Number**  **(10^6^/ejaculate)** | **Progressive Motility**  **(%)** | **Non Progressive Motility**  **(%)** | **Abnormal Forms**  **(%)** | **Leukocytes**  **(10^6^/ml)** | **Germ**  **cells** | **Epithelial**  **cells** |
| --- | --- | --- | --- | --- | --- | --- | --- | --- | --- | --- | --- |
| **#10** | A | 6 | ND | 80 | 80 | 50 | / | 90 | 0.7 | present | rare |
| **#20** | A | 58 | ND | / | / | / | / | / | 0.2 | rare | rare |
| **#71** | A | 31 | ND | 10 | 20 | 40 | / | 93 | 0.6 | present | numerous |
| **#74** | B | 16, 31, 58 | ND | 15 | 25.5 | 20 | 5 | 97 | 1.0 | present | absent |
| **#126** | B | 53 | ND | 48 | 163.2 | 35 | 5 | 94 | 1.2 | numerous | absent |
| **#175** | A | 18 | ND | / | / | / | / | / | 0.2 | absent | absent |
